# Analyses of circRNA and mRNA Profiles in Vogt–Koyanagi–Harada Disease

**DOI:** 10.3389/fimmu.2021.738760

**Published:** 2021-12-22

**Authors:** Jia Shu, Guannan Su, Jun Zhang, Zhangluxi Liu, Rui Chang, Qingfeng Wang, Peizeng Yang

**Affiliations:** The First Affiliated Hospital of Chongqing Medical University, Chongqing Key Laboratory of Ophthalmology, Chongqing Eye Institute, Chongqing Branch of National Clinical Research Center for Ocular Diseases, Chongqing, China

**Keywords:** VKH disease, CD4^+^ T cell, circular RNA, competing endogenous RNA network, RNA-seq

## Abstract

Recent studies revealed that circular RNAs (circRNAs) are important in numerous biological process and involved in autoimmune diseases. However, their role in Vogt–Koyanagi–Harada (VKH) disease, a classical autoimmune disease, is not yet known. This research aimed to study the expression profile of mRNAs, microRNAs (miRNAs) and circRNAs and investigate the influence of circRNAs on the pathogenesis of VKH disease. We identified circRNAs, miRNAs, and mRNAs expression profiles in CD4^+^ T cells between 4 VKH patients and 3 healthy controls using the whole-transcriptome sequencing (RNA-seq) technique. We discovered that a total of 5088 mRNAs, 451 circRNAs and 433 miRNAs were differently expressed. The GO and KEGG pathway enrichment analyses were performed for significantly differentially expressed circRNAs and mRNAs. GSEA was conducted for all mRNAs. The functional enrichment suggested that the inflammatory response, the adaptive immune response, NF-kappa B signaling pathway, Th17 cell differentiation, Th1 and Th2 cell differentiation and T cell receptor signaling pathway were associated with VKH disease. In addition, based on the immune-related genes we screened, the circRNA-miRNA-mRNA ceRNA network was analyzed and constructed. Ten differently expressed mRNAs (LAT, ZAP70, ITK, ICOS, RASGRP1, PAG1, PLCG1, PRKCQ, LCK, CARD11) and 5 differently expressed circRNAs (hsa_circ_0033144, hsa_circ_0000233, hsa_circ_0000396, hsa_circ_0001924, hsa_circ_0001320) were selected to be validated by Real-time qPCR (RT-qPCR). The results of RT-qPCR turned out to be consistent with RNA-seq data. Further analysis showed that hsa_circ_0001320 and hsa_circ_0001924 may serve as crucial candidate marker genes of VKH disease. These results reveal that circRNAs may have a crucial immunomodulatory function in the pathophysiological process of VKH disease.

## Introduction

VKH disease is a systemic autoimmune disorder characterized by bilateral granulomatous uveitis with systemic involvement such as meningeal irritation, auditory dysfunction, vitiligo, alopecia, and poliosis (1–3). Based on the previous studies from our group, VKH disease accounts for 12.7-15.9% of total uveitis patients (4, 5). It is generally accepted that VKH disease is an autoimmune disorder mediated by immune response against melanin associated antigens (1). Studies indicate that the overreaction of Th1 and Th17 cells is involved in pathogenesis of this disease (6–9). However, the regulation of both cell populations in this disease remains unclear.

Recent studies show that ncRNAs are important in the pathogenesis of autoimmune disease (10–13). According to length and shape, these ncRNAs can be divided into miRNAs, lncRNAs and circRNAs (14). It has been shown that a number of miRNAs are able to enhance the production of pro-inflammatory immune cells through upregulation of Th1 and Th17 signaling pathway (15). CircRNAs can serve as miRNA sponges to regulate the downstream mRNAs indirectly by carrying out the function of competing endogenous RNA (ceRNA) (16–18). A recent report reviewes the role of some “star circRNAs” in systemic lupus erythematosus (SLE) (19, 20). Study on Sjögren’s syndrome (SS) shows that hsa_circRNA_104121 and hsa_circRNA_045355 are associated with disease activity index and laboratory parameters (21). Circ_0005402 and circ_0035560 are reported to be expressed at a low level in multiple sclerosis (MS) patients and could be used as biomarkers of the disease (22). A recent study showed that ncRNAs is important in uveitis (23). However, there was no report about the role of circRNAs and ceRNA networks in VKH disease.

In this study, we conducted the RNA-seq to identify the expression profile of circRNAs and constructed the circRNAs-miRNAs-mRNAs regulatory network in VKH disease. These results suggest that circRNAs and the ceRNA regulatory network are involved in the pathogenic mechanism of VKH disease, which provide a new insights into the underlying mechanism of this disease.

## Materials and Methods

### Participants

This study included 10 VKH patients presenting with an active intraocular inflammation and 9 healthy controls. Four VKH patients and 3 healthy controls were included in the whole-transcriptome sequencing stage, and an additional 6 VKH patients and 6 healthy controls were included in the subsequent RT-qPCR verification test. The patients did not receive any immunosuppressive agents for at least 7 days, while the healthy controls did not receive any medicine within a month. The diagnosis of this disease followed the criteria proposed by an international committee on nomenclature (24), and the modified criteria reported by our team (25). These VKH patients appeared evident intraocular inflammation with detailed information listed in **Table S1**. All VKH patients enrolled were diagnosed in the uveitis center of the First Affiliated Hospital of Chongqing Medical University from January 2020 to May 2021. The study received the approval of the Ethics Committee of the First Affiliated Hospital of Chongqing Medical University and all procedures followed the tenets of the Declaration of Helsinki, and written informed consents were obtained from all participants.

### Isolation of CD4^+^T Cells

The CD4^+^ T cells used in this study were purified from peripheral blood mononuclear cells (PBMCs) using the human CD4 microbeads (Miltenyi Biotec, Bergisch Gladbach, Germany). To fully isolate PBMCs from fresh heparinized blood, we used density-gradient centrifugation provided by Ficoll-Hypaque. We stored the CD4^+^ T cells at -80° C for subsequent experiments.

### RNA-Seq Analysis

Total RNA of CD4^+^ T cells was extracted using TRIzol reagent (Invitrogen, San Diego, CA, United States) following manufacturer’s protocol. The quality of total RNA samples was measured using Nanodrop 2000 (Thermo Fisher Scientific). Agilent 2100 bioanalyzer (Agilent Technologies, SantaClara, CA, USA) was used to test the RNA integrity. RNA Integrity Number (RIN) was used as a screening criteria, with samples RIN ≥ 7 to be subjected to the subsequent analysis. The ribosomal RNA was digested from the total RNA using the Ribo-Zero Gold kit (Illumina, USA). The libraries were then constructed and sequenced on the Illumina Hiseq2500 platform, the150-bp/125-bp paired-end reads were produced. The original sequencing data were obtained from Illumina platform. All of the RNA-seq and analyses were performed by OEbiotech (Shanghai, China). Considering the possible bias that may exist, the original data were pre-processed by Trimmomatic to ensure the validity and robustness (26).

The differentially expressed genes between controls and VKH patients were determined by DEseq software, and then screened according to the fold change and the results of significance test. The criteria of fold change > 2 or <0.5 (|log2(fold-change) | >1), with p-value < 0.05 and false discovery rate (FDR) <0.05, were used to identify differentially expressed genes between these groups.

### RNA Preparation and RT-qPCR

Total RNA was extracted from CD4^+^ T cells using TRIzol (Invitrogen, San Diego, CA, United States), and reversely transcribed by Prime Script RT reagent kit (Takara, Kusatsu, Japan) following the manufacturer’s protocol. RT-qPCR was performed with ABI 7500 Real-Time PCR System (Applied Biosystems, California, US) using SYBR Green Kit (Takara, Kusatsu, Japan). All primers were synthesized by Sangon (Shanghai, China). **Table 1** lists all the used sequences of primers. The 2^-△△CT^ method was used to normalize the relative expression of mRNAs and circRNAs to GAPDH.

**Table 1:** Primers used for quantitative real time PCR.

RNA	Forward Primer	Reverse Primer
GAPDH	GGGTGTGAACCATGAGAAGT	CAGTGATGGCATGGACTGTG
LAT	GATGAGGACGACTATCACAACCC	GAAGGCACTGTCTCGGATGC
ZAP70	CTGGAGCTATGGGGTCACCA	CAGGCTGTAGTAACAGGCTCG
ITK	TAAATACCCGTTTCAGGTGGTG	GGTTGGATCATATTGGGCACAG
ICOS	ACAACTTGGACCATTCTCATGC	TGCACATCCTATGGGTAACCA
RASGRP1	TGGAAACCTGTGTCGAAGTAAC	ACTCCTCCATAGTGTCTGTCAAG
PAG1	TTCAGCCGTTCAGTTACTAGCC	TGGACTTCCTCGTAATGCTGC
PLCG1	GGAAGACCTCACGGGACTTTG	GCGTTTTCAGGCGAAATTCCA
PRKCQ	GCAAAAACGTGGACCTCATCT	CAAAGAAGCCTTCCGTCTCAAA
LCK	TCTGCACAGCTATGAGCCCT	GAAGGAGCCGTGAGTGTTCC
CARD11	AACCTTCCAGGAGCGGTACTA	GTAGCGCATGGCTAAGTTGTA
hsa_circ_0033144	AAAGGCATCTGTCCCAAG	CGTCTTCTTCGAGGATGG
hsa_circ_0000233	TTGTTATTAAGGCTGATGTT	CGATTATTTGTAGGCTTAGT
hsa_circ_0000396	CTAAGCAACGCCATTATG	ACACAGTAGACACTCAATA
hsa_circ_0001924	GAAGCCAAGAATTGGGAG	CCATATTTCTTCCACTCGC
hsa_circ_0001320	GTGAAGCAGTGTGCGAAGA	CTTGAGGTGTCATCATAGCCA

### Pathway Enrichment and Biological Function Analysis

The functions of the parental genes of significantly differential expressed circRNAs and mRNAs regulated by circRNA-miRNA were examined by GO and KEGG pathway analyses. All mRNAs were analyzed using GSEA. In the GO database, the function of genes was separated into three parts, namely biological process (BP), molecular function (MF) and cellular component (CC). Using the GO database, we explored what functions the target genes were mainly related to in these three parts. KEGG is a pathway-related database that supplies annotation of disease-associated pathways for significantly enriched genes. GSEA is an assessment method that calculates whether the difference between pre-identified sets of genes was statistically significant in healthy controls and VKH patients. We used the STRING database to obtain the protein–protein interaction (PPI) network of differentially mRNAs involved in pathways associated with immune inflammation (27). The PPI network was constructed by Cytoscape software (version 3.7.2). Gene Ontology (http://www.geneontology.org), the KEGG database (http://www.genome.jp/kegg), GSEA database (http://www.gsea-msigdb.org) and STRING database (https://www.string-db.org/) were used for our analysis.

### Construction of the circRNA-miRNA-mRNA Network

The expression levels of differentially expressed miRNAs, mRNAs and circRNAs were used for co-expression analysis. We calculated the p-value and Pearson’s correlation coefficient for miRNA-target pairs. Pairs with negative correlation containing a Pearson’s correlation coefficient > 0.9 and p value < 0.05 were used for further analysis. The binding sites of these miRNA-mRNA pairs and miRNA-circRNA pairs were predicted using the miRANDA program, and the predicted pairs were selected for further analysis. The predicted pairs were obtained by taking the intersection of the two analyses above. Subsequently, these predicted pairs were chosen to conduct ceRNA score calculations based on the ceRNA formula (28). Cytoscape software (Version 3.7.2) was used to construct the circRNA-miRNA-mRNA interaction network.

### Statistical Analysis

All the data were displayed with the mean ± standard deviation (SD). Student’s t test was used to determine if a significant difference exists between two comparing groups. We considered p-value < 0.05 to be statistically significant. All statistical analyses were carried out using GraphPad Prism version 8.0 (GraphPad, San Diego, CA, USA).

## Results

### Overview of circRNAs Profiles

We conducted whole-transcriptome sequencing on the Illumina Hiseq2500 platform. After normalization, the datasets for the mRNAs/miRNAs/circRNAs of 7 samples were distributed nearly similar and no abnormal expression was found as shown by box plot in **Figure S1**. Using principal component analysis (PCA), we concluded that samples in the same group showed a high degree of similarity (**Figure S2**).

**Figure 1:**
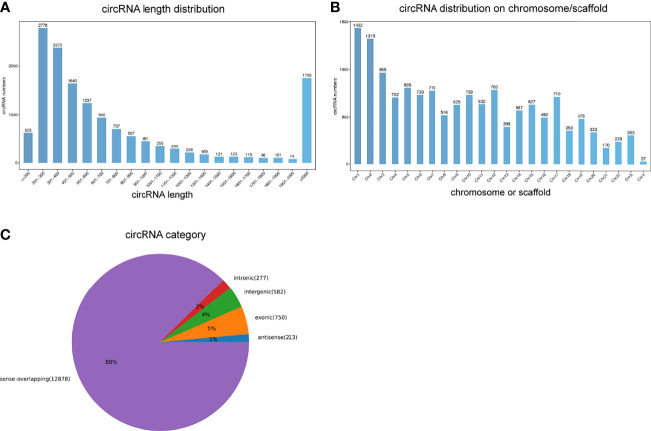
Overview of circRNAs profiles. **(A)** The length distribution of circRNAs. **(B)** The chromosome distribution of circRNAs. **(C)** The gene structure distribution of circRNAs.

An average of 89 million and 86 million clean reads were identified in VKH patients and healthy controls. We used those clean reads to identify circRNAs. A majority of them was mapped to the reference genome. A total of 17400 circRNAs were selected as candidates. Most circRNAs sequences ranged from 201 nt to 300 nt according to the distribution of circRNA sequence lengths (**Figure 1A**). These differentially expressed circRNAs were widely distributed on chromosomes, mostly on the chromosomes 1, 2, and 3 (**Figure 1B**). The positional distribution of circRNAs on genome revealed that the majority of circRNAs were sense-overlapping (88%) with only a minor proportion of them being intergenic (4%) or intronic (2%) (**Figure 1C**).

### Identification of Differentially Expressed mRNAs, miRNAs and circRNAs

**Figure 2:**
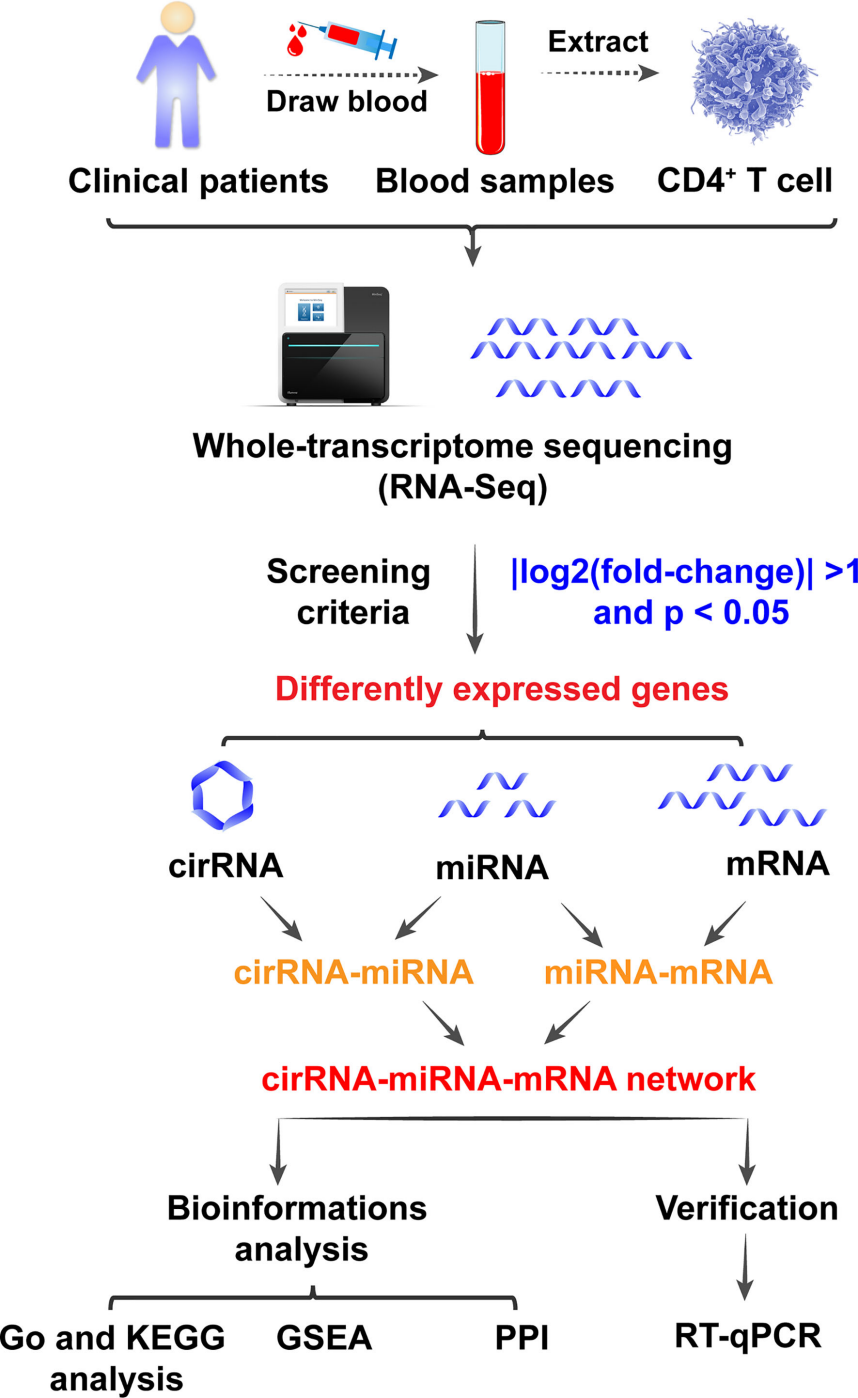
Graphical abstract.

**Figure 3:**
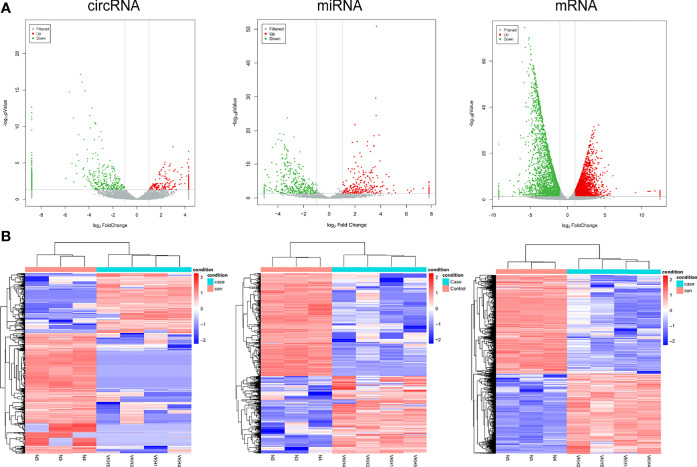
Expression Profiles of circRNAs, miRNAs, and mRNAs in active VKH patients and healthy controls. **(A)** Cluster analysis of differentially expressed circRNAs, miRNAs and mRNAs. Red indicates high expression and blue represents low expression **(B)** The volcano plot of differentially expressed circRNAs, miRNAs and mRNAs.

The differentially expressed mRNAs, miRNAs and circRNAs were identified following the criteria of |log2(fold-change)| >1 and p < 0.05 as stated above. The screening process is shown in **Figure 2**. According to the screening criterion, there were 451 differentially expressed circRNAs (165 upregulated and 286 downregulated), 433 differentially expressed miRNAs (182 upregulated and 251 downregulated), and 5088 mRNAs (2289 upregulated and 2799 downregulated) between controls and VKH patients. The data of differentially expressed mRNAs, miRNAs and circRNAs are displayed using Volcano plot (**Figure 3A**) and heat map (**Figure 3B**) respectively. The mRNAs, miRNAs, and circRNAs with the most significant upregulation and downregulation in VKH disease are listed in **Table 2**. The most upregulated mRNA, miRNA, and circRNA were EPGN (log2FoldChange=7), hsa-miR-509-3p (log2FoldChange=8), and hsa_circ_0102898 (log2FoldChange=4). The most downregulated mRNA, miRNA, and circRNA were ALOX15B (log2FoldChange=-9), hsa-miR-1910-5p (log2FoldChange=–5), and hsa_circ_0087960 (log2FoldChange=-6). **Tables S2–S4** **list** the top 10 mRNAs, miRNAs and circRNAs that are upregulated and downregulated.

**Table 2:** The most significantly upregulated and downregulated circRNAs, miRNAs and mRNAs.

DE RNAs	Total	Upregulated	Downregulated	The Most Upregulated [log2(fold-change)]	The Most Downregulated [log2(fold-change)]
circRNA	451	165	286	hsa_circ_0102898 (4)	hsa_circ_0087960 (–6)
miRNA	433	182	251	hsa-miR-509-3p (8)	hsa-miR-1910-5p (–5)
mRNA	5088	2289	2799	EPGN (7)	ALOX15B (–9)

### Functional and Pathway Enrichment Analysis

**Figure 4:**
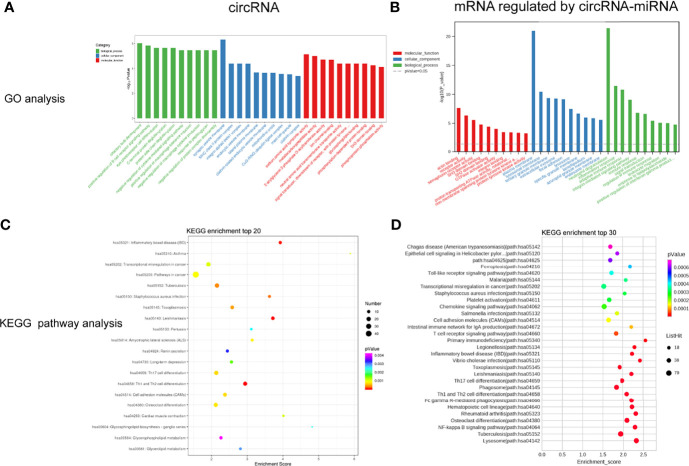
GO enrichment analysis and KEGG pathway analysis for the differently expressed circRNAs **(A, C)** and mRNAs regulated by circRNA-miRNA **(B, D)**.

To study the biological function of circRNAs in VKH disease, the GO and KEGG databases were used to analyze the parent genes of these circRNAs. GO analysis, as shown in **Figure 4A**, which indicated that the parent genes of differentially expressed circRNAs were mainly enriched in positive regulation of B cell receptor signaling pathway, positive regulation of macrophage tolerance induction, eye photoreceptor cell differentiation and negative regulation of macrophage cytokine production. KEGG analysis displayed a large number of parent genes that were mainly enriched in Th1 and Th2 cell differentiation, inflammatory bowel disease (IBD), adhesion molecules (CAMs), toxoplasmosis and Th17 cell differentiation and cell (**Figure 4C**). **Tables S5, S6** shows the enriched pathways and functions of the differentially expressed circRNAs.

**Figure 5:**
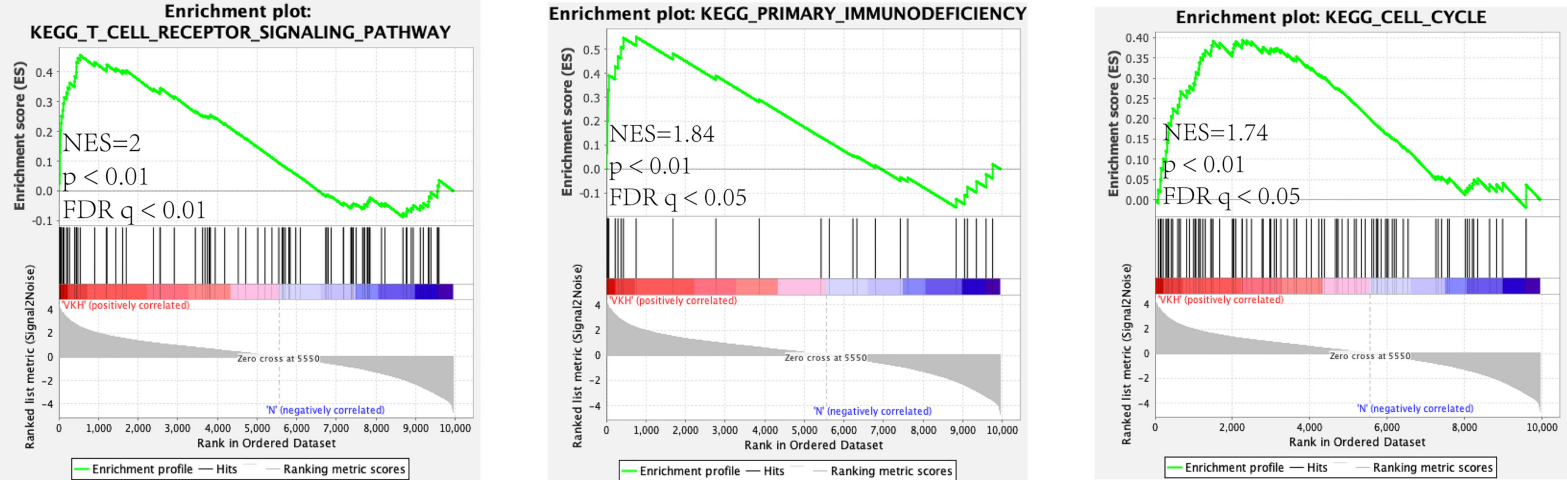
Gene set Enrichment analysis (GSEA) for mRNA.

As mentioned above, we analyzed and constructed the circRNA-miRNA-mRNA networks. The mRNAs in the network were selected to conduct further analysis. As displayed in **Figure 4B**, GO analysis displayed that the differentially expressed mRNAs in the circRNA-miRNA-mRNA network were associated with neutrophil degranulation, inflammatory response, regulation of immune response, adaptive immune response and integrin-mediated signaling pathway. KEGG pathway analysis revealed top 30 enriched pathways among these differentially expressed mRNAs, including NF-kappa B signaling pathway, rheumatoid arthriti, Th17 cell differentiation, Th1 and Th2 cell differentiation and toll-like receptor signaling pathway **(****Figure 4D**). **Tables S7, S8** lists the enriched pathways and functions of the differentially expressed mRNAs in the circRNA-miRNA-mRNA network. In addition, we performed Gene Set Enrichment Analysis (GSEA) on all mRNAs of the RNA-seq data, and discovered that these mRNAs were mainly related to T cell receptor signaling pathway, primary immunodeficiency pathway and cell cycle pathway (**Figure 5**).

A further study was performed on these differentially expressed mRNA according to the PPI network analysis. As indicated in **Figure 7A**, 15 hub genes were identified in the PPI network including IL2, TNF, IL10, IL6, CTLA4, FOXP3, CD28, IL13, CD40, CD40LG, TBX21, IL2RA, IL12RB1, GATA3 and STAT4.

### Validation of Differentially Expressed mRNAs and circRNAs by RT-qPCR

**Figure 6:**
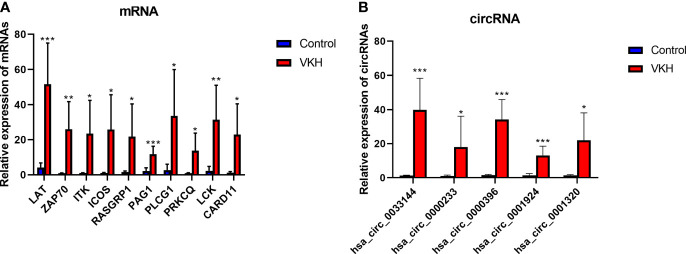
Validation of differentially expressed mRNAs and circRNAs in VKH group and control group. **(A)** The expression levels of 10 mRNAs detected by RT-qPCR of 6 VKH patients and 6 healthy controls. **(B)** The expression levels of 5 circRNAs detected by RT-qPCR in CD4^+^ T cells of 6 VKH patients and 6 healthy controls. The data are presented as the mean ± SD. *P < 0.05; **P < 0.01; ***P < 0.001.

To verify the RNA-seq data, we selected some circRNAs and mRNAs and measured their expression levels by RT-qPCR. Ten immune-related mRNAs (LAT, ZAP70, ITK, ICOS, RASGRP1, PAG1, PLCG1, PRKCQ, LCK, CARD11) selected according to bioinformatics analysis and 5 circRNAs (hsa_circ_0033144, hsa_circ_0000233, hsa_circ_0000396, hsa_circ_0001924, hsa_circ_0001320) randomly selected were validated using RT-qPCR **(****Figures 6A** **, B**). The experiment showed results similar to the RNA-seq data.

### Construction of the circRNA-miRNA-mRNA ceRNA Regulatory Network

**Figure 7:**
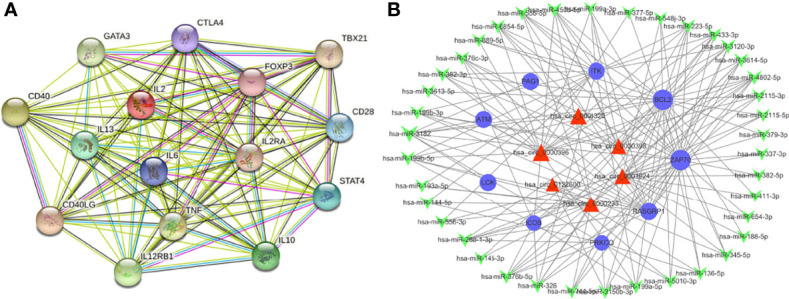
Overview of the networks. **(A)** The PPI network of differently expressed mRNAs based on the STRING database. **(B)** Construction of circRNA-miRNA-mRNA ceRNA network based on the selected vital genes related to immune and inflammation. The ceRNA regulatory network includes 9 mRNAs, 39 miRNAs and 6 circRNAs. The blue circular nodes represent mRNAs, the red triangular nodes indicate circRNAs, the green arrow nodes denote miRNAs.

Previous studies showed that circRNAs can sponge miRNAs and thereby indirectly regulate the expression of mRNAs. MiRanda program was used to speculate target genes of these miRNAs. They were then compared with the differentially expressed genes identified following the criteria stated above. To investigate the role of the ceRNA network in pathogenic mechanism of VKH disease, we selected a series of enriched mRNAs closely related to immune and inflammatory pathways based on GO, KEGG and GSEA analysis, which appeared most frequently in immune and inflammation-related pathways, including LAT, ZAP70, ITK, ICOS, RASGRP1, PAG1, PLCG1, PRKCQ, LCK, CARD11, BCL2, ATM. The cirRNA-miRNA-mRNA networks were built up depending on the target mRNAs. These networks included 69 circRNAs, 210 miRNA and 12 mRNAs. They were used to build up a total of 6212 circRNA-miRNA-mRNA targeting axes. Since the constructed ceRNA network is too complicated to display, we filtered out the interested genes to construct a simplified network, which helps us to better analyze its functions and to screen out crucial circRNAs. (**Figure 7B**). A total of 109 circRNA-miRNA-mRNA axis were built up including 9 mRNAs, 39 miRNAs and 6 circRNAs. As shown in the figure, hsa_circ_0001924, hsa_circ_0001320, hsa_circ_0000398, hsa_circ_0000396, hsa_circ_0000233, hsa_circ_0122600 in the ceRNA network may regulate these immune-related mRNAs through the ceRNA mechanism, thus participating in the pathophysiological process of VKH disease. Based on relevant literatures and databases, hsa_circ_0001924 and hsa_circ_0001320 were screened out as potential candidate genes in VKH disease. **Table S9** shows all circRNA-miRNA-mRNA relationship pairs.

## Discussion

In this study, we investigated the expression profile of mRNAs, miRNAs, and circRNAs in active VKH patients using the RNA-seq technology. We found that a total of 5088 mRNAs 433 miRNAs and 451 circRNAs were differently expressed in VKH patients compared to controls based on previous screening criteria. Based on the ceRNA screening strategy and bioinformatics analysis, the immune-related ceRNA regulatory networks in VKH disease were analyzed and predicted. The KEGG pathway analysis, GO enrichment analysis and GSEA showed that these RNAs largely involved in the adaptive immune response, Th1 and Th2 cell differentiation, Th17 cell differentiation, NF-kappa B signaling pathway and T cell receptor signaling pathway.

Previous studies concerning circRNAs expression are almost performed on PBMCs (29–31). As Th1 and Th17 cells, two important subsets of CD4^+^ T cells, play crucial roles in the development of VKH disease, we focused on the aberrant expression of circRNAs in these CD4^+^T cells in this study. The sequencing results showed 451 differentially expressed circRNAs in active VKH patients, suggesting their possible involvement in this disease. Importantly, based on bioinformatics analysis and validation experiment, we identified that hsa_circ_0001320 and hsa_circ_0001924 may serve as candidate genes for VKH disease. Our results are, by and large, similar to that in keloid reported by Zhang et al. (32). They show that the hsa_circ_0001320 has an aberrantly higher expression in this disease. An aberrantly lower expression of hsa_circ_0001924 is reported in idiopathic pulmonary fibrosis (IPF) (33), known as an immune-driven disease. The expression of hsa_circ_0001924 in IPF seems to be paradoxical to our results whereby a higher expression of this circRNA was observed in active VKH disease. This difference could be explained by the nature of these two diseases. IPF is caused by a deficiency of Th1 cells (34), whereas VKH disease is mediated by overaction of Th1 and Th17 cells. Our results collectively suggest that circRNAs play a role in these autoimmune diseases, although other circRNAs may also be involved and the exact mechanisms may be somewhat different among these diseases.

It has been well established that miRNAs can result in gene silencing by binding to mRNA, whereas circRNAs can compete with miRNAs and further regulate the expression of mRNAs. This mode of regulation is defined as the CeRNA mechanism (35–37). In fact, RNA-seq technology has been applied to the experimental autoimmune uveitis (EAU), the classic animal model of autoimmune uveitis, to identify a series of miRNAs that were enriched in T cell receptor signaling pathway, chemokine signaling pathway, etc (38). However, the circRNA profile in EAU model has not been investigated. This technique has also been applied to study the ncRNA network in a variety of other autoimmune diseases. Kachamakova-Trojanowska et al. identified the expression profile of miRNAs in pulmonary sarcoidosis, showing that the target genes of miRNAs were enriched in Ras signaling, which is the downstream of T cell receptor (39). Zurawska et al. reported that the circRNA profile of multiple sclerosis revealed links to B-cell function (15), which is in accordance with our findings. Zhang et al. found that hsa_circ_0123190 can act as a ceRNA to regulate APLNR expression by sponging hsa-miR-483-3p in lupus nephritis. Yang et al. reported the circRNA_09505 could serve as a miR-6089 sponge to regulate inflammation *via* miR-6089/AKT1/NF-kappa B axis in collagen-induced arthritis (CIA) model (31).

In our study, RNA sequencing of miRNAs and mRNAs were simultaneously performed to address the expression profile of these RNAs and their potential relationship. A further experiment was designed to validate the RNA-seq data. A total of 10 mRNAs and 5 circRNAs were selected to be validated by RT-qPCR, and the experimental results were consistent with those of RNA-seq results. We further established ceRNA network based on the sequencing results. Interestingly, the differentially expressed circRNAs identified in VKH disease are mainly involved in Th17 cell differentiation, positive regulation of B cell receptor signaling pathway, NF-kappa B signaling pathway and Th1 and Th2 cell differentiation. These results are consistent with those reported previously (1, 40). The mRNAs regulated by circRNA-miRNA are associated with T cell receptor signaling pathway, inflammatory response, adaptive immune response, toll-like receptor signaling pathway and NF-kappa B signaling pathway. These pathways are also proven to be involved in the pathogenic mechanism of other immune-related diseases. A recent study reports that circRNAs, which appeared in the Th17 cell differentiation pathway, may participate in the pathogenic mechanism of chronic obstructive pulmonary disease (COPD), a chronic inflammatory immune disease (41). The differentially expressed circRNAs identified during Eimeria tenella (E. tenella) infection were reported to be involved in NF-kappa B signaling pathway and B cell receptor signaling pathway (42). The level of circRNAs expression can influence the expression of mRNAs associated with the toll-like receptor signaling pathway in acute myeloid leukemia (43). CircRNAs play a role in the treatment of non-alcoholic steatohepatitis (NASH) with traditional Chinese medicine through T cell receptor signaling pathway (44). All of these results suggest that these aberrantly expressed circRNAs may exert their effects through immune signaling pathways and inflammatory immune response, therefore contributing to development of VKH disease. On the other hand, the results of GSEA revealed the potential involvement of immunodeficiency in VKH disease. VKH disease is uncommon in immunocompromised patients such as acquired immunodeficiency disease syndrome (AIDS) caused by the human immunodeficiency virus (HIV) virus. At present, there is only one report of VKH disease in HIV patients, in which the author proposed that this may be a coincidental occurrence or there might exist potential association since both VKH disease and HIV/AIDs target CD4^+^ T cells (45).

There are some limitations in this study. First, the sample size is relatively small. As consider that drugs might have potential impact on the RNA transcriptome, we only included patients who had not received any immunosuppressive agents for at least 7 days, which limited the number of patients enrolled in the study. Second, we only studied the active VKH patient group and the control group, but did not include other uveitis entities as positive control. Whether these differential genes are specific biomarkers of VKH disease still awaits future investigation. Third, further adding inactive VKH patients as a control group would be of great significance to show whether the differentially expressed RNAs identified in this study are specific to active VKH patients or could be generalized to all VKH patients. In addition, with the rapid development and application of high-throughput sequencing technology, increasing circRNAs were identified and annotated (46, 47). However, the functional characterization of circRNAs is still at its early stage (48), and there is a lack of quantitative method to systematically evaluate which circRNAs have the potential to serve as biomarkers. These issues are expected to be clarified in future study.

In conclusion, this is the first study to investigate the comprehensive expression profiles of mRNAs miRNAs and circRNAs in VKH disease. Our findings may provide new insights into the pathogenic mechanism and pathological process of VKH disease. Furthermore, differentially expressed circRNAs identified in the current study may be potentially used for the study on biomarker, diagnosis as well as the evaluation of treatment in this disease.

## Data Availability Statement

The datasets presented in this study can be found in online repositories. The names of the repository/repositories and accession number(s) can be found in the article/**Supplementary Material**.

## Ethics Statement

The studies involving human participants were reviewed and approved by The Ethics Committee of the First Affiliated Hospital of Chongqing Medical University. The patients/participants provided their written informed consent to participate in this study.

## Author Contributions

PY and JS conceived the idea and designed the study. JS and ZL collected the sample. JS performed all the experiments. JS and JZ analyzed the data. RC and QW checked data. JS wrote the manuscript. PY and GS interpreted data and revised the manuscript. All authors contributed to the article and approved the submitted version.

## Funding

This study was supported by National Natural Science Foundation Key Program (81930023), Natural Science Foundation Major International (Regional) Joint Research Project (81720108009), Chongqing Outstanding Scientists Project (2019), the Chongqing Chief Medical Scientist Project (2018), Chongqing Key Laboratory of Ophthalmology (CSTC, 2008CA5003) and Chongqing Science & Technology Platform and Base Construction Program (cstc2014pt-sy10002).

## Conflict of Interest

The authors declare that the research was conducted in the absence of any commercial or financial relationships that could be construed as a potential conflict of interest.

## Publisher’s Note

All claims expressed in this article are solely those of the authors and do not necessarily represent those of their affiliated organizations, or those of the publisher, the editors and the reviewers. Any product that may be evaluated in this article, or claim that may be made by its manufacturer, is not guaranteed or endorsed by the publisher.
