# Supplementary material for: Analyses of circRNA and mRNA Profiles in Vogt–Koyanagi–Harada Disease
Source: Front Immunol. 2021 Dec 22;12:738760. doi: 10.3389/fimmu.2021.738760 (PMC8727692; doi:10.3389/fimmu.2021.738760)
Supplement: Supplementary file 1 [file DataSheet_1.docx]

Supplementary Material

# Supplementary Figures and Tables

## Supplementary Figures

**
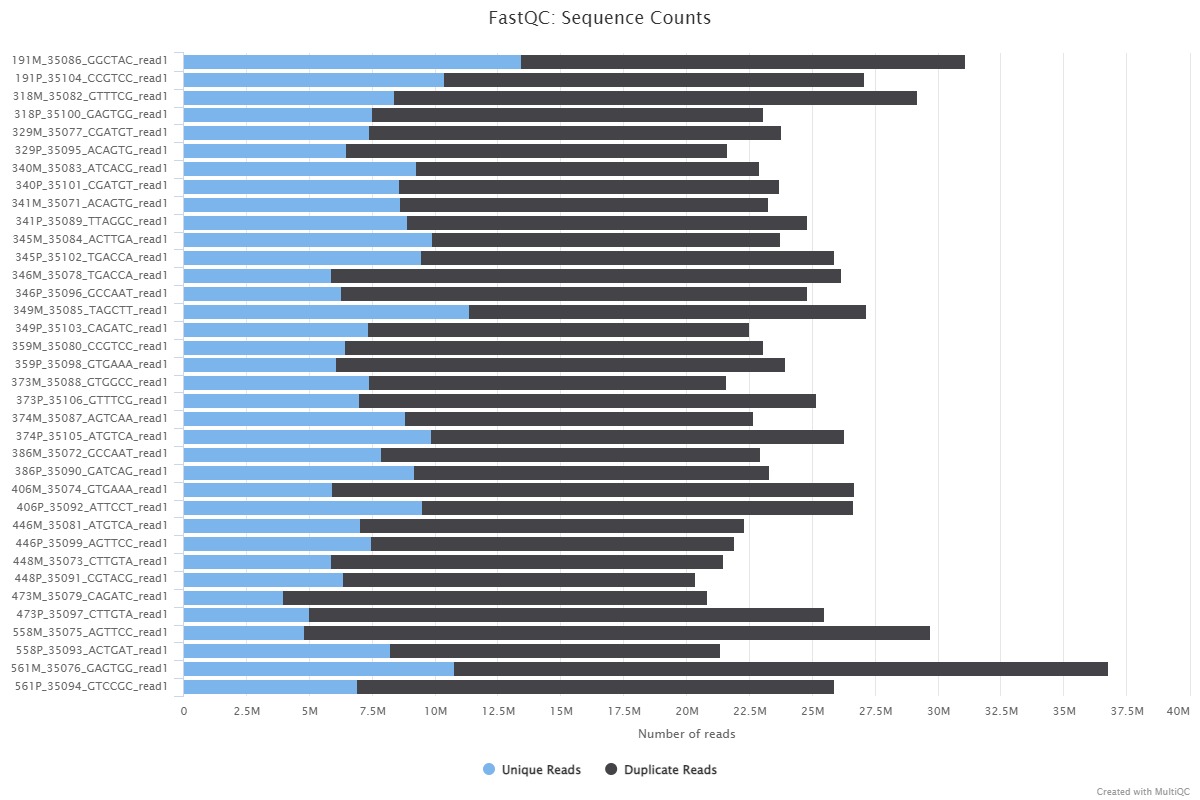
**

**Supplementary Figure 1.** Sequence counts for each sample showing unique (blue) and duplicate (black) reads. Duplicate read counts (in blue) are an estimate only.

**
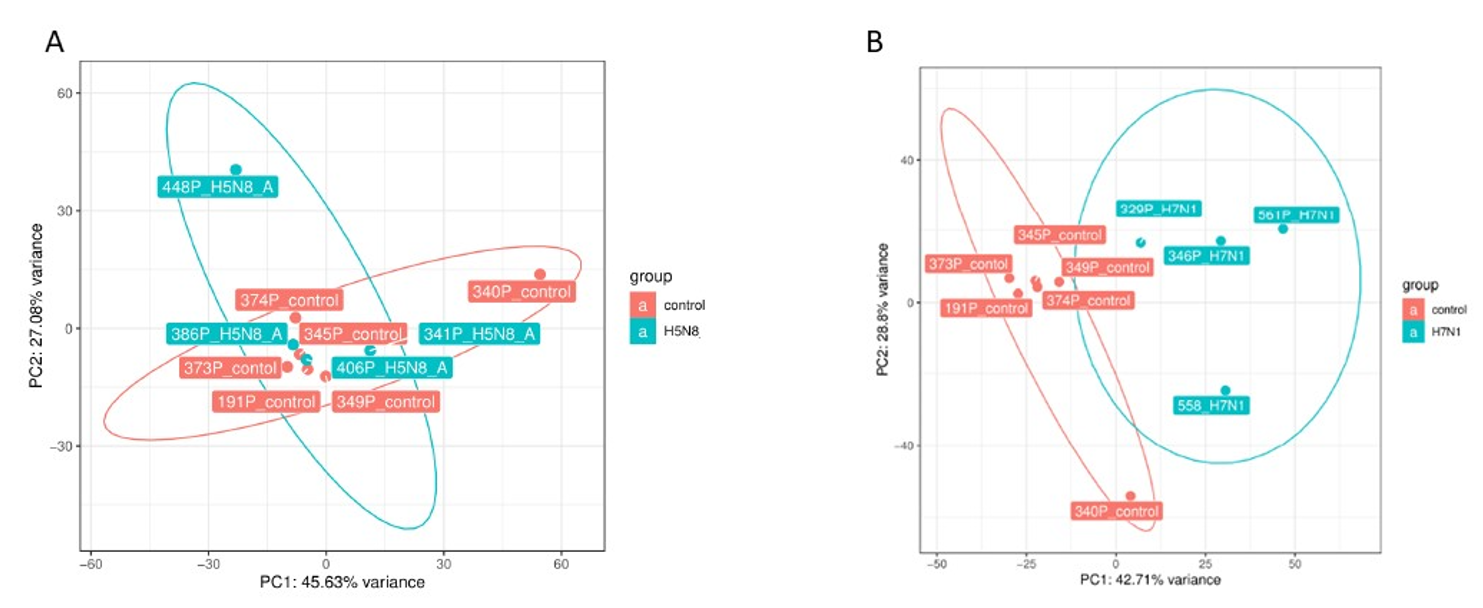
**

**Supplementary Figure 2.** Principal component analysis plot on variance stabilizing transformed (VST) counts of lung samples collected from resistant H5N8 inoculated chickens and control chickens (A), and from susceptible H7N1 inoculated chickens and control chickens (B). Used to discard 448, 340, and 558 (lung sample) chickens as outliers.


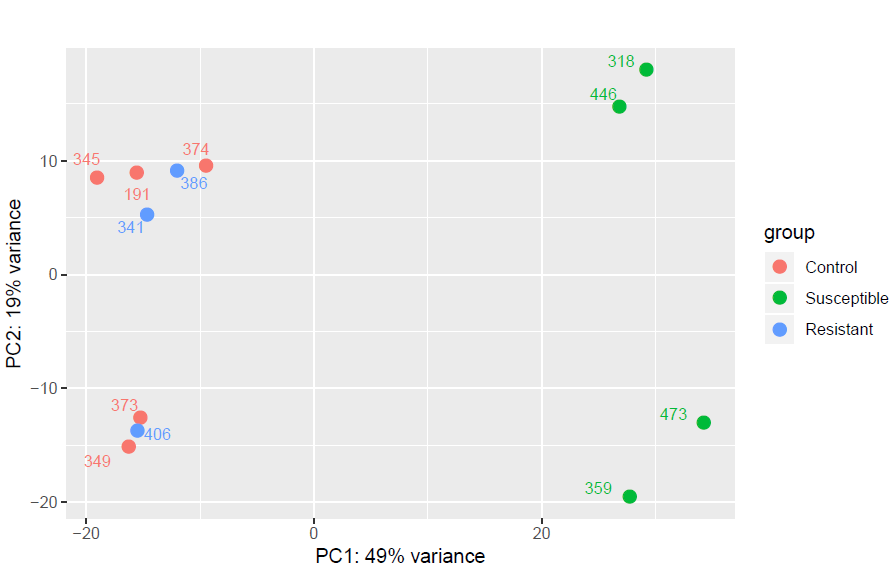


**Supplementary Figure 3.** Principal component analysis plot on variance stabilizing transformed (VST) counts of lung samples collected from susceptible and resistant H5N8 inoculated chickens and control chickens.

**
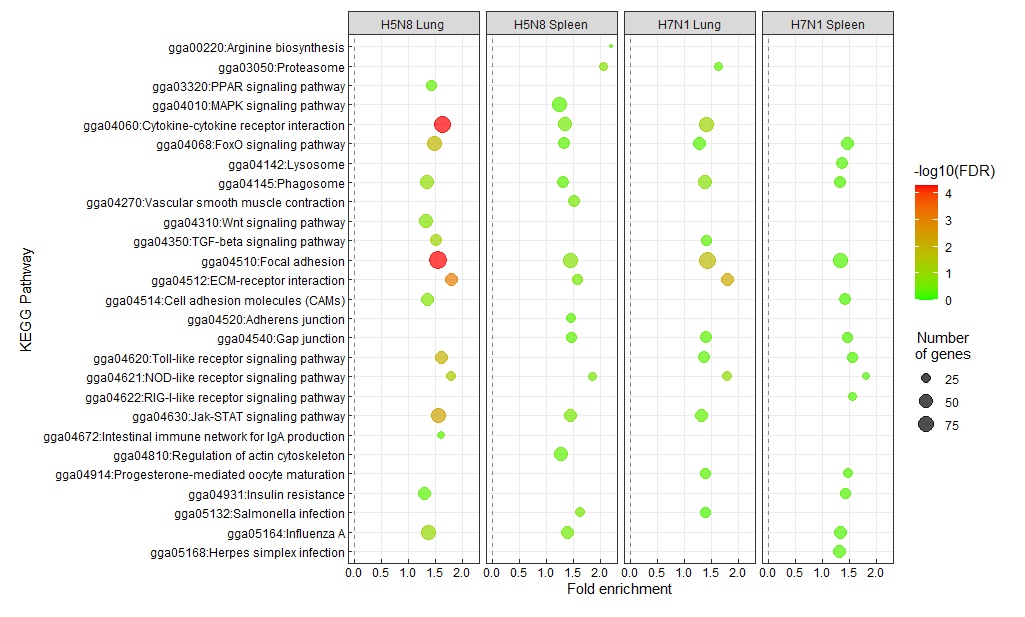
**

**Supplementary Figure 4.** Enriched GO terms obtained from KEGG for DEGs in lungs or spleens collected at 3 dpi from susceptible H5N8 or H7N1 inoculated chickens. The most representative and significant biological processes are represented and are sorted by fold enrichment. The dot size indicates the number of DEGs associated with the biological process. The dot color indicates the significance of the enrichment (-log10 (FDR-corrected P-values)).


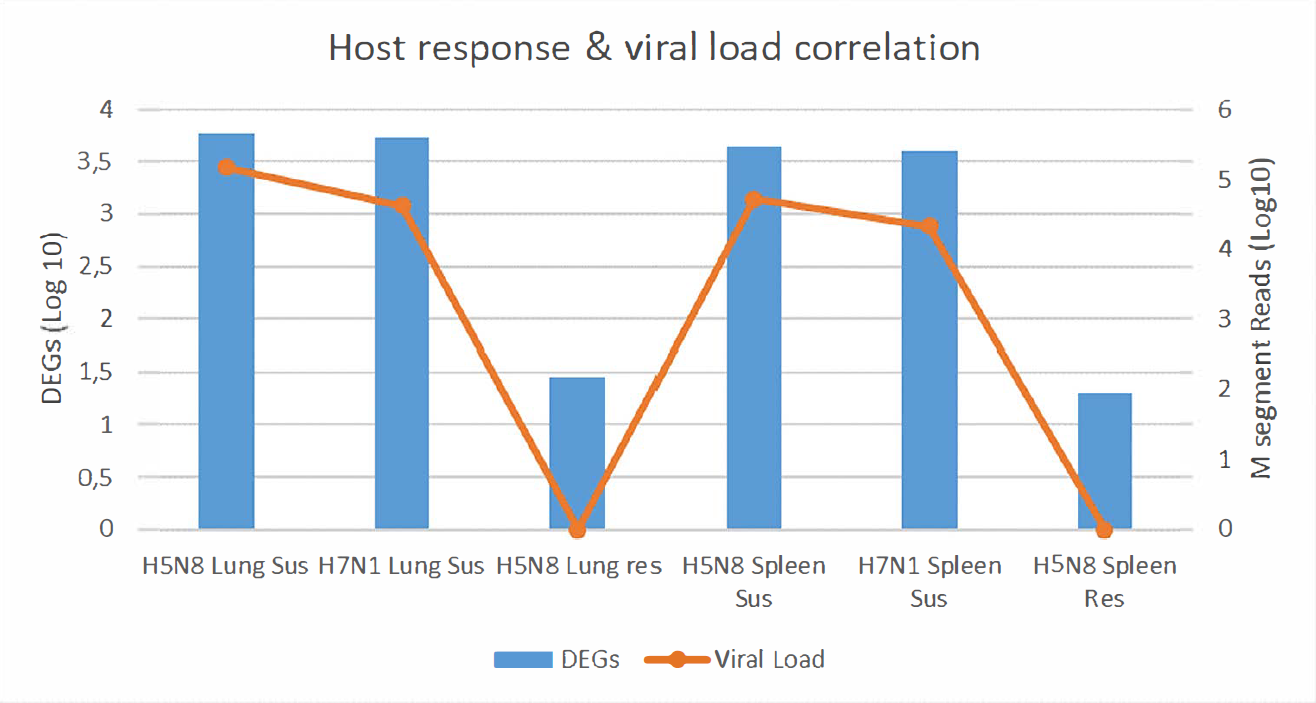


**Supplementary Figure 5**. Number of DEGs (bars, left y-axis) and viral transcripts (line, right y-axis) identified by RNA-Seq in lungs and spleens collected at 3 dpi from susceptible (Sus) or resistant (Res) H5N8 or H7N1 inoculated chickens.


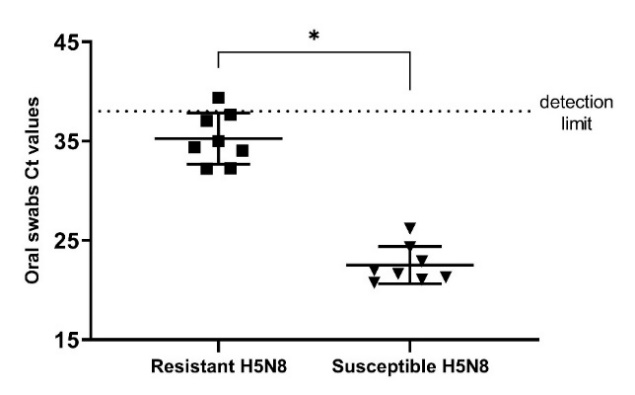


**Supplementary Figure 6**. Quantitative RT-PCR of M gene from 3 dpi oral swabs obtained from resistant and susceptible H5N8 inoculated chickens. For each group the mean and standard deviation are (*) P≤ 0.05; unpaired two-tailed t-test. The dotted line represents the limit of detection (Ct 38).


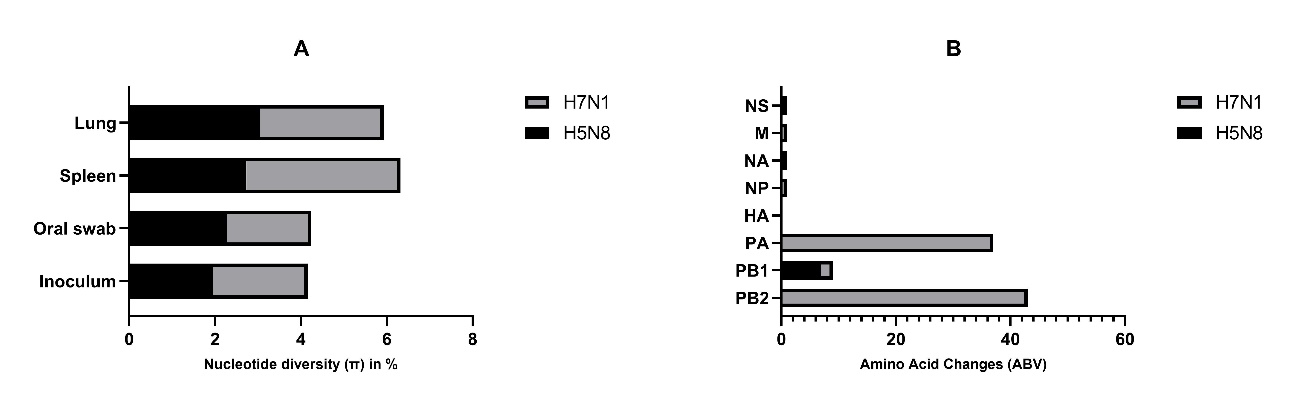


**Supplementary Figure 7**. Stacked bars showing amino acid changes in absolute numbers in each segment. H7N1 viruses are represented by gray colour and H5N8 by black colour.

## 1.2 Supplementary Tables

**Supplementary Table 1.** Classification as resistant (RES), susceptible (SUS) or control (CON) of the samples (lung (LNG), spleen (SPL) or allantoic fluid) and breeds (Empordanesa (EMP), Penedesenca (PENED), Catalana del Prat (C. PRAT), Flor d’Ametller (F. AMET), Castellana negra (C. NEGRA), Euskal oiloa (E. OILOA), Broiler, and specific pathogen free (SPF)) used for this study. 1a. RNA-Seq sample selection and classification. 1b. Quasi-species sample selection and classification. 1c. *PLAU* validation sample selection and classification. 1d. Summary of all the samples.

1a.

| RNA-Seq sample selection | | | | | |
| --- | --- | --- | --- | --- | --- |
| *VIRUS* | *CHICKEN ID* | *BREED* | *DPI* | *SAMPLE* | *GROUP* |
| H5N8 | 341 | C.NEGRA | 3 | LNG, SPL | RES |
|  | 386 | BROILER | 3 | LNG, SPL | RES |
|  | 406 | EMP | 3 | LNG, SPL | RES |
| H7N1 | 558 | E.OILOA | 3 | SPL | SUS |
|  | 561 | E.OILOA | 3 | LNG, SPL | SUS |
|  | 329 | BROILER | 3 | LNG, SPL | SUS |
|  | 346 | BROILER | 3 | LNG, SPL | SUS |
| H5N8 | 473 | F.AMET | 3 | LNG, SPL | SUS |
|  | 359 | C.NEGRA | 3 | LNG, SPL | SUS |
|  | 446 | SPF | 3 | LNG, SPL | SUS |
|  | 318 | E.OILOA | 3 | LNG, SPL | SUS |
| CONTROL | 345 | BROILER | 3 | LNG, SPL | CON |
|  | 349 | E.OILOA | 3 | LNG, SPL | CON |
|  | 191 | EMP | 3 | LNG, SPL | CON |
|  | 374 | SPF | 3 | LNG, SPL | CON |
|  | 373 | C.PRAT | 3 | LNG, SPL | CON |

1b.

| Quasi-species sample selection | | | | | |
| --- | --- | --- | --- | --- | --- |
| *VIRUS* | *CHICKEN ID* | *BREED* | *DPI* | *SAMPLE* | *GROUP* |
| H7N1 | 558 | E. OILOA | 3 | ALLANTOIC FLUID | SUS |
|  | 329 | BROILER | 3 | ALLANTOIC FLUID | SUS |
|  | 346 | BROILER | 3 | ALLANTOIC FLUID | SUS |
|  | 959 | PENED | 3 | ALLANTOIC FLUID | SUS |
| H5N8 | 359 | C.NEGRA | 3 | ALLANTOIC FLUID | SUS |
|  | 446 | SPF | 3 | ALLANTOIC FLUID | SUS |
|  | 318 | E. OILOA | 3 | ALLANTOIC FLUID | SUS |
|  | 319 | E. OILOA | 3 | ALLANTOIC FLUID | SUS |
|  | 200 | EMP | 3 | ALLANTOIC FLUID | SUS |
|  | 433 | C.PRAT | 3 | ALLANTOIC FLUID | SUS |
|  | 467 | F. AMET | 3 | ALLANTOIC FLUID | SUS |
|  | 471 | F. AMET | 3 | ALLANTOIC FLUID | SUS |

1c.

| *PLAU* qPCR sample selection | | | | | |
| --- | --- | --- | --- | --- | --- |
| *VIRUS* | *CHICKEN ID* | *BREED* | *DPI* | *SAMPLE* | *GROUP* |
| H7N1 | 950 | EMP | 3 | LNG | RES |
| H5N8 | 423 | PENED | 3 | LNG | RES |
|  | 442 | C.PRAT | 3 | LNG | RES |
|  | 387PN | BROILER | 3 | LNG | RES |
| H7N1 | 954 | EMP | 3 | LNG | SUS |
|  | 946 | PENED | 3 | LNG | SUS |
|  | 572 | F.AMET | 3 | LNG | SUS |
|  | 558 | E.OILOA | 3 | LNG | SUS |
|  | 561 | E.OILOA | 3 | LNG | SUS |
|  | 346 | BROILER | 3 | LNG | SUS |
| H5N8 | 433 | C.PRAT | 3 | LNG | SUS |
|  | 367 | C.NEGRA | 3 | LNG | SUS |
| CONTROL | 349 | E.OILOA | 3 | LNG | CON |
|  | 353 | PENED | 3 | LNG | CON |
|  | 399 | PENED | 3 | LNG | CON |
|  | 373 | C.PRAT | 3 | LNG | CON |
|  | 378 | F.AMET | 3 | LNG | CON |
|  | 379 | F.AMET | 3 | LNG | CON |

1d.

| Chicken Id | Virus | Breed | Group | RNA seq | QSP | qPCR |
| --- | --- | --- | --- | --- | --- | --- |
| 341 | H5N8 | C.NEGRA | RES | X |  |  |
| 386 | H5N8 | BROILER | RES | X |  |  |
| 406 | H5N8 | EMP | RES | X |  |  |
| 558 | H7N1 | E.OILOA | SUS | X | X | X |
| 561 | H7N1 | E.OILOA | SUS | X |  | X |
| 329 | H7N1 | BROILER | SUS | X | X |  |
| 346 | H7N1 | BROILER | SUS | X | X | X |
| 473 | H5N8 | F.AMET | SUS | X |  |  |
| 359 | H5N8 | C.NEGRA | SUS | X | X |  |
| 446 | H5N8 | SPF | SUS | X | X |  |
| 318 | H5N8 | E.OILOA | SUS | X | X |  |
| 345 | Control | BROILER | CON | X |  |  |
| 349 | Control | E.OILOA | CON | X |  | X |
| 191 | Control | EMP | CON | X |  |  |
| 374 | Control | SPF | CON | X |  |  |
| 373 | Control | C.PRAT | CON | X |  | X |
| 959 | H7N1 | PENED | SUS |  | X |  |
| 319 | H5N8 | E. OILOA | SUS |  | X |  |
| 200 | H5N8 | EMP | SUS |  | X |  |
| 433 | H5N8 | C.PRAT | SUS |  | X |  |
| 467 | H5N8 | F. AMET | SUS |  | X |  |
| 471 | H5N8 | F. AMET | SUS |  | X |  |
| 950 | H7N1 | EMP | RES |  |  | X |
| 423 | H5N8 | PENED | RES |  |  | X |
| 442 | H5N8 | C.PRAT | RES |  |  | X |
| 387 | H5N8 | BROILER | RES |  |  | X |
| 954 | H7N1 | EMP | SUS |  |  | X |
| 946 | H7N1 | PENED | SUS |  |  | X |
| 572 | H7N1 | F.AMET | SUS |  |  | X |
| 433 | H5N8 | C.PRAT | SUS |  |  | X |
| 367 | H5N8 | C.NEGRA | RES |  |  | X |
| 353 | Control | PENED | CON |  |  | X |
| 399 | Control | PENED | CON |  |  | X |
| 378 | Control | F.AMET | CON |  |  | X |
| 379 | Control | F.AMET | CON |  |  | X |

**Supplementary Table 2.** Primers used for quantitative real time PCR analysis of chicken mRNAs.

| **target** | **Primers 5´-3´** |
| --- | --- |
| RPL12 Rev | GGCCCGTGTTATCTCAGAGG |
| RPL12 Rev | GGATCCCAAAGAGACGAGCG |
| ACTB Fwd | AGCGAACGCCCCCAAAGTTCT |
| ACTB Rev | AGCTGGGCTGTTGCCTTCACA |
| YWHAZ Fwd | AGGAGCCGAGCTGTCCAATG |
| YWHAZF Rev | CTCCAAGATGACCTACGGGCTC |
| PLAU | qGgaCED0031197 Bio RAD |
| Il1B Fwd | GGGCATCAAGGGCTACAA |
| Il1B Rev | CTGTCCAGGCGGTAGAAGAT |
| VCAM1 Fwd | GAGATTGTTTGTGTGGCCAGATT |
| VCAM1 Rev | TGGAGATGCATTAATGGAAGTATTTT |
|  |  |

**Supplementary Table 3.** List of DEGs identified from lungs in resistant birds.

| Upregulated DEGs | Downregulated DEGs |
| --- | --- |
| *ANAPC10* | *LOC107051977* |
| *LOC112529948* | *ADGRG1* |
| *HEMGN* | *TNFRSF1A* |
| *HBM* | *LOC112532108* |
| *R3HDM4* | *DGKG* |
| *HBA1* | *CYTH4* |
| *EPB42* | *PGF* |
| *RRAD* | *ITGBL1* |
| *LOC112531211* | *SLC24A4* |
| *RHCE* | *FGF14* |
| *C8ORF88* | *VCAM1* |
| *HBBA* | *LOC107056992* |
| *BLOC1S6* | *ADRB3* |
| *MFSD2B* | *LOC107055113* |
| *RHAG* | *PRLHRL* |
| *NT5C3A* | *PLAU* |
| *SLC25A37* |  |
| *NARF* |  |
| *MIR147-1* |  |
| *LOC107054431* |  |
| *C4H4ORF54* |  |
| *SAT1* |  |
| *ANKRD54* |  |

**Supplementary Table 4.** Number of *PLAU* reads by lung sample in the resistant (RES), susceptible (SUS) and control groups from the RNA-Seq analysis.

| CHICKEN ID | BREED | GROUP | *PLAU* READS |
| --- | --- | --- | --- |
| 386 | BROILER | RES | 184 |
| 406 | EMP | RES | 196 |
| 341 | C.NEGRA | RES | 177 |
| 318 | E.OILOA | SUS | 1355 |
| 359 | C.NEGRA | SUS | 1680 |
| 446 | SPF | SUS | 1620 |
| 473 | F.AMET | SUS | 759 |
| 374 | SPF | CONTROL | 1061 |
| 373 | C.PRAT | CONTROL | 716 |
| 191 | EMP | CONTROL | 529 |
| 345 | BROILER | CONTROL | 499 |
| 349 | E.OLIOA | CONTROL | 724 |

Supplementary Table 5: Amino acid changes at the quasi-species level in each segment and virus.

| H7N1 | |  | H5N8 | |
| --- | --- | --- | --- | --- |
| Segment | Amino acid changes |  | Segment | Amino acid changes |
| M | T181A |  | NA | Y113F |
| NP | K98R |  | NS | S266I |
| PA | Y59E P65S F71L R73K R74H L75R Y76F T78I A80E L86M L90V C93S V94I R572K F573I T575M H578G M582R R584S Q593E V598A C601S V612F I618T F625P V627G I628V R631G T632S P634G T638R V649L F652S Y656E |  | PB1 | K82D R86D *88A *89A D105H D110N L115A |
| PB1 | M571R S770C |  |  |  |
| PB2 | G60D R61K M67I F68P D69E G70R I71N V77L R79S C81T K82N K84A *85G P86S G87D L89V A91V A92S T93P V94L K95A K96V Q104P D120E A122V T132P Q134H *138Q A635S H654P K657N N658Y I662T I663K A673G S675L T690V R702K |  |  |  |
